# Severe Heart Failure After Fetal Loss and COVID-19: A Diagnostically Challenging Case With Complicated Timing

**DOI:** 10.7759/cureus.36866

**Published:** 2023-03-29

**Authors:** Roy Lim, Rachel R Johnson, Brian Denney, Lina Zalikha

**Affiliations:** 1 Internal Medicine, Mount Sinai Hospital, Chicago, USA; 2 General Medicine, American University of the Caribbean School of Medicine, Cupecoy, SXM; 3 General Medicine, Cebu Velez General Hospital, Cebu City, PHL; 4 General Medicine, Ross University School of Medicine, Two Mile Hill, BRB

**Keywords:** coronavirus disease 2019, heart failure, peripartum cardiomyopathy, ejection fraction, heart failure with reduced ejection fraction, ppcm, peripartum dilated cardiomyopathy, heart failure patients, covid-19

## Abstract

Coronavirus disease 2019 (COVID-19), initially recognized to cause respiratory system complications, has been found to also affect the cardiovascular system leading to myocardial damage and subsequently causing heart failure. Peripartum cardiomyopathy, though an uncommon condition, may also manifest as heart failure toward the end of pregnancy. This atypical case highlights the potential diagnostic overlap between COVID-19 heart failure and peripartum cardiomyopathy. At this point, there is no recommended algorithm used to distinguish one disease from another.

## Introduction

Coronavirus disease 2019 (COVID-19) is an infectious disease caused by the severe acute respiratory syndrome coronavirus 2 (SARS-CoV-2) virus, first reported from Wuhan, China in 2019. It mainly presents as a respiratory system disease; however, some cases produce a variety of signs and symptoms, from flu to a wide range of cardiovascular diseases. Some reports noted significant cardiac damage in patients with COVID-19, even in the absence of clinical features of respiratory disease that can also lead to heart failure, including in young patients with no pertinent cardiac history [1].

Peripartum cardiomyopathy (PPCM) is a rare cause of heart failure that typically occurs during the last month of pregnancy or up to five months postpartum [2]. According to the American Heart Association (AHA), about 1,000 to 1,300 women develop PPCM in the United States each year [2]. This condition can be devastating with some reports showing mortality rates of 6-10% [2]. This condition usually does not occur before 36 weeks, making PPCM a complicated case. In a patient with PPCM, either in the pregnancy or postpartum period, guideline-directed medical therapy (GDMT) can be utilized. Angiotensin-converting enzyme (ACE) inhibitors, angiotensin-receptor blockers (ARBs), and mineralocorticoid receptor antagonists are contraindicated in pregnancy but can be given during the postpartum period. These medications are teratogenic. Angiotensin receptor neprilysin inhibitors (sacubitril-valsartan) need to be avoided in pregnancy, and there is still a lack of data about their safety during breastfeeding; however, beta-blockers, such as metoprolol and bisoprolol, can be administered in both periods [3]. Implantable cardioverter-defibrillator (ICD) has not been advised, especially for patients with newly diagnosed PPCM, given the high rate of full recovery of left ventricular function during/after GDMT [4]. One study showed a significantly increased risk of relapse in patients with left ventricular ejection fraction (LVEF) < 55% [5].

Herein, we present a case of a female with a past medical history of intrauterine fetal demise (IUFD) at 32 weeks age of gestation (AOG) and a recent COVID-19 infection admitted for shortness of breath. Electrocardiogram (EKG) demonstrated a new left bundle branch block (LBBB). Troponin I and B-type natriuretic peptide (BNP) were elevated at the initial workup. Further workups, such as a chest computed tomography (CT) scan, showed cardiomegaly with no evidence of pulmonary embolism, and the echocardiography showed a reduced LVEF of 10-15%. The patient was started on sacubitril-valsartan, carvedilol, and furosemide. She underwent a coronary angiogram, which showed no restriction in the blood flow. The patient was then discharged and scheduled for follow-ups as an outpatient to optimize her GDMT.

Our patient's case is unusual, given her new onset heart failure during the peripartum period following a course of COVID-19 infection. She presented three weeks postpartum following IUFD and induced delivery at 32 weeks. This case illustrates an overlap of two diseases, which are also the cause of heart failure. Adding to the challenge was the lack of data describing the typical timing of heart failure following COVID-19 diagnosis. Furthermore, the likelihood of developing PPCM earlier than 36 weeks following preterm fetal loss is unknown. It is important to differentiate between these two rare etiologies of heart failure because the expected prognosis may vary for each. Additionally, while the immediate management may be identical, deducing the underlying etiology is pertinent to provide evidence-based patient guidance on the safety of future pregnancies.

## Case presentation

The case involves a 37-year-old female, G5P4 (4014), with a history of IUFD at 32 weeks AOG and a recent COVID-19 infection prior to admission. She underwent genetic amniocentesis at 23 weeks AOG, which revealed trisomy 18 abnormality.

The patient in this case was unvaccinated against COVID-19. Medical history showed that her husband and children had gotten sick with COVID-19 a week before she got her first COVID-19 symptoms. She had a fever for three days, followed by sore throat, diffuse myalgias, and productive cough, which lasted for about a week. The patient sought a consult at another healthcare institution a week after and was informed that her baby had died approximately four to five days prior and thus, labor was induced. About three weeks later, she began feeling fatigued and dyspneic with substernal pleuritic chest pain and associated back pain upon exertion, which prompted a consult at our institution.

The patient presented with orthopnea, bilateral leg edema, and dyspnea on exertion, especially after walking half block. She denied any febrile episodes, chills, palpitations, cough, paroxysmal nocturnal dyspnea, dizziness, abdominal pain, nausea, vomiting, or changes in the urine. The patient denied the use of any alcoholic beverages, tobacco, or illicit drugs. She was then admitted for further evaluation and management.

Vital signs were within normal limits except for a blood pressure of 137/95 mmHg and a heart rate of 125 beats per minute. Auscultation revealed bibasal fine crackles, unremarkable cardiovascular findings with regular S1 and S2, no murmurs, rubs, or gallops, no jugular vein distention, and bilateral 1+ lower extremity edema.

**Figure 1:**
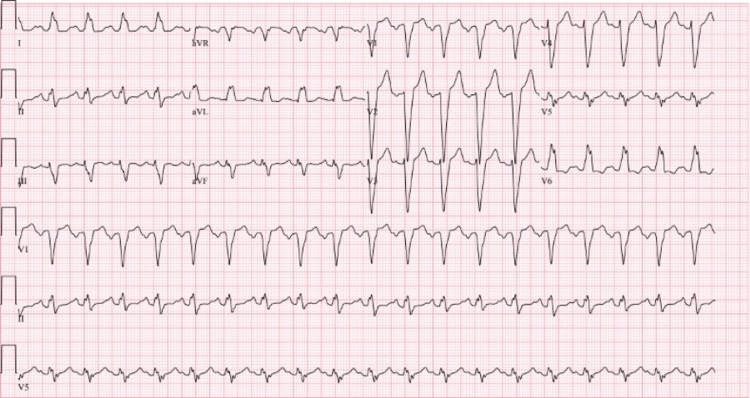
EKG showing sinus tachycardia, left atrial enlargement, and new left bundle branch block

Figure 1 shows the EKG of the patient, which revealed sinus tachycardia, left atrial enlargement, and new LBBB with a high QTc of 510. No prior EKG was available for comparison.

Table 1 shows the pertinent laboratory results on admission, which were significant for leukocytosis at 14.2 103/UL, hemoglobin of 13.4 g/dL, no electrolyte abnormalities, troponin was slightly elevated around 30 ng/mL, which downtrend to 23 ng/mL, BNP was 441 pg/mL, and COVID-19 test was negative.

**Table 1:** Laboratory workup results on admission This table shows an elevated B-type natriuretic peptide of 441 pg/mL and an elevated troponin of 30 ng/mL, likely concerning demand ischemia from fluid overload.

Test	Result	Reference value
Sodium	138	133-134 meq/L
Potassium	4.1	3.5-5.2 meq/L
Chloride	106	98-107 meq/L
Calcium	9.4	8.6-10.3 mg/dL
Fasting blood glucose	97	70-99 mg/dL
Blood urea nitrogen	17	7-25 mg/dL
Creatinine	0.8	0.6-1.2 mg/dL
Total protein	7.2	6.4-8.9 mg/dL
Troponin I	30	0-0.04 ng/mL
B-type natriuretic peptide	441	<100 pg/mL
Complete blood count		
White blood cell count	11.9	4.5-11 10^3^/UL
Red blood cell count	4.51	4.0-5.28 10^6^/UL
Hemoglobin	13.3	12.0-16.0 g/dL
Hematocrit	38.5	36-46%
Platelet count	368	150-450 10^3^/UL

**Figure 2:**
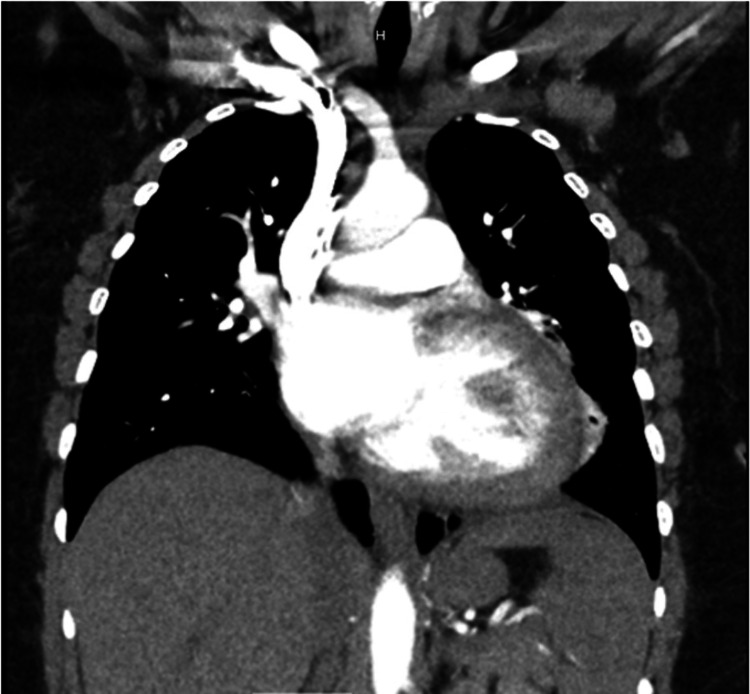
Chest CT scan (coronal view) This view of the chest CT scan demonstrated that the patient has cardiomegaly.

**Figure 3:**
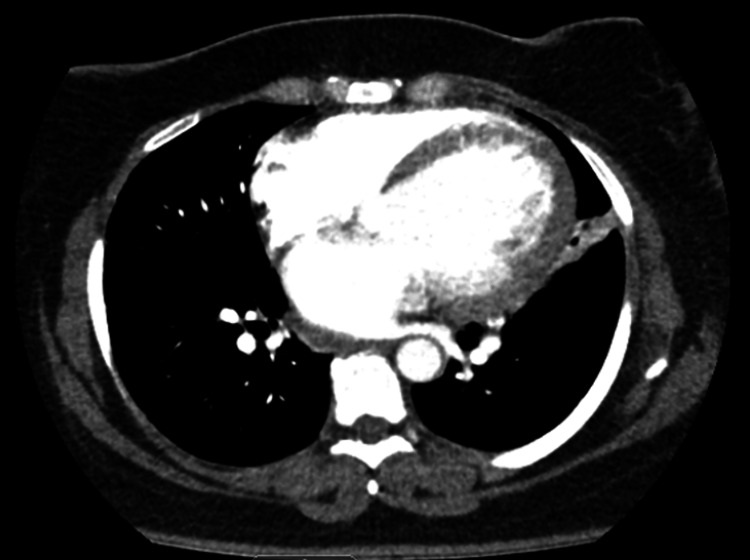
Chest CT scan (axial view) This view shows ground glass opacities on top of the heart enlargement.

Figure 2 shows the patient’s chest CT scan, which revealed moderate cardiomegaly, and Figure 3 shows subtle diffuse nonspecific peripheral ground glass opacities, several mildly prominent mediastinal lymph nodes, and no evidence of pulmonary emboli.

**Figure 4:**
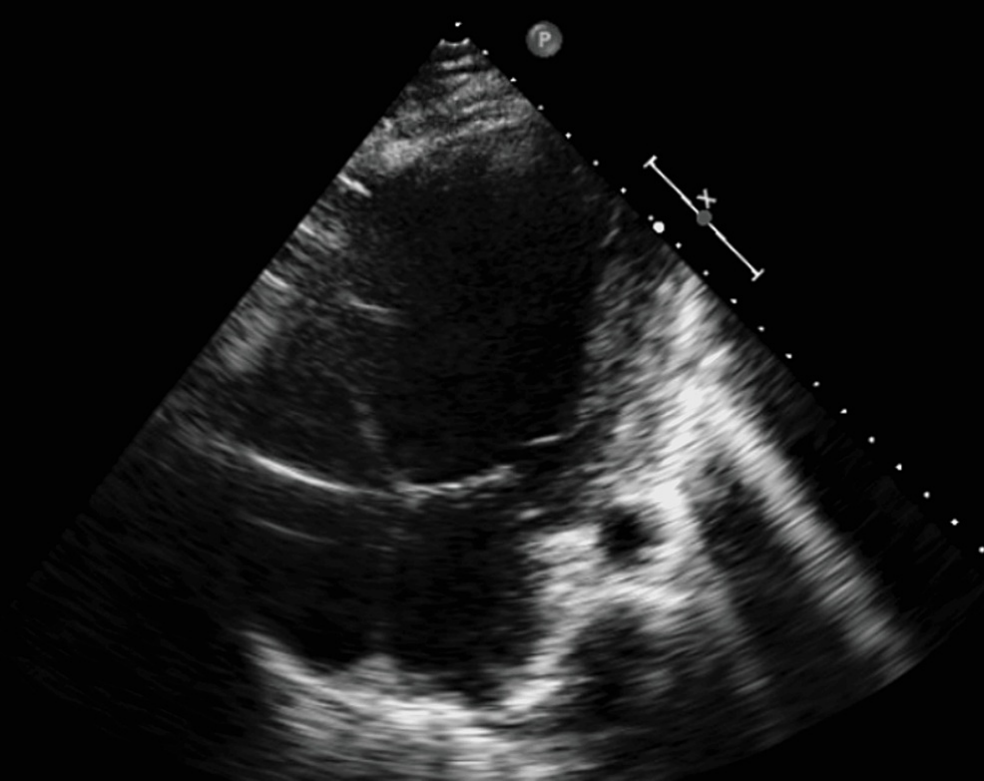
Two-dimensional echocardiogram This view of echo shows that there is a left heart strain signifying a severe left ventricular systolic dysfunction. Echo also reveals an ejection fraction of 10-15% demonstrating that there is an acute decompensation of heart failure.

An echocardiogram (Figure 4) revealed severely reduced left ventricular systolic function with an ejection fraction (EF) of 10-15%, severe diffuse hypokinesis with no identifiable regional variations, and mild mitral regurgitation with a posteriorly directed jet likely secondary to left ventricle cavity dilatation causing tethering of the mitral valve leaflets.

Sacubitril-valsartan, carvedilol, and furosemide were started. The patient underwent cardiac catheterization showing no significant coronary artery disease. Telemetry over her admission showed no significant events.

The patient was discharged on the sixth hospital day after clinical improvement and returned as an outpatient for GDMT. The patient followed up after four days, still with mild bipedal edema but with notable improvement from the last visit. The patient’s current status was explained to her, including the importance of strictly adhering to her medical regimen. According to current recommended guidelines, patients who develop PPCM have a higher risk of developing it again during subsequent pregnancies. Hence, the patient was counseled on the medical risks of another pregnancy.

During the follow-up, an echocardiogram was repeated, showing a similar EF of 10-15%. The patient underwent a biventricular ICD. For about four months after, an echocardiogram was done again and showed an improved EF of 40-45%.

## Discussion

PPCM is a rare cause of heart failure occurring either at the end of pregnancy or within months after delivery [3]. Reports estimating the incidence of PPCM in the United States vary widely, ranging from one case per 1,000 to 4,000 live births. The etiology of PPCM is still unclear and previously healthy women may still be affected.

In our patient’s case, the timing of the presentation is complicated. At 31 weeks of gestation, she experienced flu-like symptoms and was diagnosed to have a mild COVID-19 infection. Around 31-32 weeks, she had an IUFD, for which she underwent an induced delivery after several days. This fetal loss was likely secondary to fetal trisomy, which was detected on amniotic fluid analysis earlier in this pregnancy. However, the timing suggests her recent COVID-19 infection may have also played a role in relation to the onset of heart failure.

The presentation could be explained by either of these etiologies separately or in combination. However, at this point, there is no clear-cut solution as to how to distinguish one disease from another. Additionally, while the immediate management may be identical, deducing the underlying etiology is important to provide patient guidance on the safety of future pregnancies.

Several associations have a different timeframe or window to consider and diagnose PPCM. According to a workshop held by the National Heart, Lung, and Blood Institute and the Office of Rare Diseases, PPCM develops in the last month of pregnancy or within five months postpartum [6]. The Heart Failure Association of the European Society of Cardiology Working Group on PPCM 2010 states that PPCM presents toward the end of pregnancy or in the months following delivery [7]. The frequency of cases occurring earlier than 36 weeks is unknown and appears to be rare.

Risk factors of our patient include hypertension and multiparity with a poor obstetrical outcome. African Americans have more risk for PPCM, while little is known about the incidence in Asians, Hispanics, and Caucasians [8]. Predisposing risk factors include older age, African American race, multiparity, multigestational pregnancies, hypertensive disorders, asthma, anemia, smoking, thyroid disorders, and prolonged usage of tocolytic agents [3,4]. PPCM’s pathogenesis remains to be unknown; however, multiple theories have been proposed [3]. PPCM is not caused by the volume overload state in the pregnancy [7]. There are a lot of theories proposing how PPCM develops, which include genetics, systemic inflammation, and autoimmune diseases. One leading mechanism is the involvement of cathepsin D and prolactin. This oxidative stress activates cathepsin D and cleaves the prolactin hormone into angiogenic subfragments, also known as 16-kD prolactin. This then induces endothelial damage [4,7]. Several studies support this theory, and there were trials adding bromocriptine, a prolactin inhibitor, to the PPCM treatment [4].

Clinical features of PPCM overlap or mimic that of normal healthy pregnancy [3]. Because of this, and given the rarity of this disease, the diagnosis is often easily overlooked [5,9]. It is crucial to assess a pregnant woman experiencing cardiovascular disease carefully. It is good to inquire about her degree of activity performance, how much she can accomplish before being out of breath, and any physical signs she has, such as cyanosis of the lips or nail beds. Another important thing to note is whether she shows signs of cough or edema. Coughing during pregnancy should always be reported because it is one of the most common symptoms of pulmonary edema that could have easily come from a cardiac pathology.

Other signs and symptoms include shortness of breath, dyspnea, cough, orthopnea, paroxysmal nocturnal dyspnea, elevated jugular venous pressure, S3, and murmur of mitral regurgitation [5,7,9,10]. PPCM is a diagnosis of exclusion [7,8]. Therefore, further diagnostic tests and imaging have to be done to rule out ischemic causes and other causes of heart failure. Cardiac biomarkers such as BNP and N-terminal proBNP (NT-proBNP) are significantly elevated as compared to normal pregnancy [4]. A threshold of <100 pg/ml for BNP and <300 for NT-proBNP can be used to rule out PPCM [3]. Echocardiography will show a left ventricular systolic dysfunction with an ejection fraction below 45% [7].

Although COVID-19 notoriously causes severe respiratory symptoms potentially leading to death, there have been reports that it dramatically affects the cardiovascular system as well by causing myocardial injury [11,12]. The SARS-CoV-2 virus is known to cause direct myocardial damage, arrhythmias, and acute coronary syndromes (ACS), which can progress to abrupt cardiac failure and shock. However, since it is new, there is not much data or studies about the cardiovascular complications brought about by this COVID-19 infection, especially its long-term outcome and prognosis.

Predisposing risk factors for COVID-19-associated heart failure include older age, hypertension, diabetes, obesity, and respiratory diseases such as asthma and chronic obstructive pulmonary disease [13]. However, COVID-19 patients can also have de novo heart failure [14]. Thus, nearly half of the heart failure patients with COVID-19 infection did not have any of the aforementioned risk factors [15].

As to how COVID-19 causes heart failure, the exact pathogenesis still remains unclear [16] and there may be varying mechanisms at play. One proposed theory is the direct attachment of the virus to the angiotensin-converting enzyme 2 (ACE2) receptors found in the heart [12,14,16]. ACE2, an aminopeptidase bound by a membrane that is extensively expressed in the cardiovascular system, is a coronavirus receptor. SARS-CoV-2 infects host cells through ACE2 receptors, leading to COVID-19-related pneumonia while also causing acute myocardial injury and chronic damage to the cardiovascular system.

In an average body, ACE plays a vital role in the renin-angiotensin-aldosterone system or the RAAS pathway. ACE is responsible for converting angiotensin I into angiotensin II. Angiotensin II is a potent vasoconstrictor, particularly of the arterioles. Although the amount of angiotensin II concentrated in the blood is usually small, its profound vasoconstrictive effects are important in certain abnormal states such as heart failure and hypovolemia. The SARS-CoV-2 infection that produces COVID-19 is caused by a protein spike that binds to ACE2 in the heart, causing cardiac issues. SARS-CoV-2 also causes acute respiratory distress syndrome (ARDS), which results in hypoxemia and the death of cardiac cells.

Other possible explanations include inflammation leading to myocarditis and atherosclerotic plaque disruption leading to myocardial infarction, ischemia-induced cardiomyocyte injury from hypoxemic respiratory failure, and coronary obstruction secondary to hypercoagulability [15,16]. Furthermore, due to coagulation dysfunction, right-sided heart failure can occur secondary to pulmonary embolism [14].

Because of these many possible mechanisms and several cardiovascular complications, the presentation of COVID-19-associated heart failure widely varies. With or without any underlying risk factors, COVID-19 patients or those recently infected patients who presented with signs and symptoms of heart failure need to have a thorough workup.

All patients should be counseled about their risks and prognosis if they consider future pregnancies soon. There is an increased risk of mortality and recurrence of the disease in patients with poor left ventricular systolic function. Pregnancy should be avoided in these patients with poor left ventricular systolic function [4]. Despite the need to avoid further pregnancies, there is also a struggle with the contraceptive method to be used. Because of the risk of thromboembolism, oral contraceptives are not recommended. The illness can advance to the point that a woman requires a heart transplant after giving birth.

Even though the management is going to be similar for both, as discussed earlier, we have come up with a table shown below that sums up several subtle points on the differences, especially on the management, outcome, and secondary prevention (Table 2). Identifying the etiology is the key part of implementing appropriate interventions.

**Table 2:** Management, outcomes, and secondary prevention of heart failure associated with PPCM and COVID-19 This table shows that even though managing PPCM and COVID-19-associated heart failure may overlap, both still have differences in the management, outcome, and ways to prevent them. PPCM: peripartum cardiomyopathy; HF: heart failure.

	PPCM	COVID-19-associated HF
Management	High threshold for cardiac devices, possible need for emergent delivery	COVID-19-specific therapy if in the window
Outcome	High chance of partial or full recovery	Appears variable; more data needed
Future targeted treatment/prevention	Bromocriptine? Counseling on future pregnancies	Vaccination

In addition to what has been said, we want to address the knowledge gap between these two diseases. As for PPCM, there is a lack of consensus with regard to its definition and clear timeframe; whether or not to include the earlier presentation prior to 36 weeks has led to confusion regarding the expected timing; enormous diversity in the gathered data about its incidence, risks, outcomes, and prognosis. Furthermore, no PPCM following a fetal loss at 32 weeks of gestation, to our knowledge, has been reported. One study mentioned that 13% of cases can present either prior to one month before delivery or more than four months postpartum [7]. There were reports from several countries wherein newly diagnosed heart failure in pregnancy occurred more than one month before delivery and five months thereafter [8]. PPCM prior to 36 weeks of gestation is rare, but cases are likely underreported.

## Conclusions

Our case of a 37-year-old female patient illustrates a challenging diagnostic scenario in which the two leading differentials are rare causes of heart failure. This illustrates an overlap of two diseases, given her new onset of heart failure during the peripartum period following a course of COVID-19 infection, which is a plausible cause of heart failure. We have pointed out knowledge gaps in the available data, which gave us difficulty in differentiating one disease from another.

We do not yet have robust data with regard to COVID-19-associated heart failure to help us disentangle and deduce how likely it is in our patient care. We do not know the likelihood or probability of a young, healthy patient developing heart failure after a COVID-19 infection. We do not yet understand the underlying mechanism of COVID-19 and PPCM and how they may or may not have contributed to this case, leading to severe heart failure in this previously healthy patient. Furthermore, while the immediate management may be identical, deducing the underlying etiology is important to provide patient guidance on the safety of future pregnancies. We also, therefore, recommend that more studies are needed. Clinicians must be able to distinguish between COVID-19-associated failure and PPCM to optimize the management and plan for secondary prevention.

## References

[1] Basu-Ray I, Almaddah NK, Adeboye A, Soos MP (2023). Cardiac Manifestations of Coronavirus (COVID-19). https://pubmed.ncbi.nlm.nih.gov/32310612/.

[2] American Heart Association (2023). American Heart Association. Peripartum cardiomyopathy. May.

[3] Jha N, Jha AK (2021). Peripartum cardiomyopathy. Heart Fail Rev.

[4] Bauersachs J, König T, van der Meer P (2019). Pathophysiology, diagnosis and management of peripartum cardiomyopathy: a position statement from the Heart Failure Association of the European Society of Cardiology Study Group on peripartum cardiomyopathy. Eur J Heart Fail.

[5] Elkayam U (2011). Clinical characteristics of peripartum cardiomyopathy in the United States: diagnosis, prognosis, and management. J Am Coll Cardiol.

[6] Pearson GD, Veille JC, Rahimtoola S (2000). Peripartum cardiomyopathy: National Heart, Lung, and Blood Institute and Office of Rare Diseases (National Institutes of Health) workshop recommendations and review. JAMA.

[7] Sliwa K, Hilfiker-Kleiner D, Petrie MC (2010). Current state of knowledge on aetiology, diagnosis, management, and therapy of peripartum cardiomyopathy: a position statement from the Heart Failure Association of the European Society of Cardiology Working Group on peripartum cardiomyopathy. Eur J Heart Fail.

[8] Isogai T, Kamiya CA (2019). Worldwide incidence of peripartum cardiomyopathy and overall maternal mortality. Int Heart J.

[9] Arany Z, Elkayam U (2016). Peripartum cardiomyopathy. Circulation.

[10] Mubarik A, Chippa V, Iqbal AM (2022). Postpartum Cardiomyopathy. App Radiol.

[11] Al-Wahaibi K, Al-Wahshi Y, Mohamed Elfadil O (2020). Myocardial injury is associated with higher morbidity and mortality in patients with 2019 novel coronavirus disease (COVID-19). SN Compr Clin Med.

[12] Babapoor-Farrokhran S, Gill D, Walker J, Rasekhi RT, Bozorgnia B, Amanullah A (2020). Myocardial injury and COVID-19: possible mechanisms. Life Sci.

[13] Haussner W, DeRosa AP, Haussner D, Tran J, Torres-Lavoro J, Kamler J, Shah K (2022). COVID-19 associated myocarditis: a systematic review. Am J Emerg Med.

[14] Bader F, Manla Y, Atallah B, Starling RC (2021). Heart failure and COVID-19. Heart Fail Rev.

[15] Long B, Brady WJ, Koyfman A, Gottlieb M (2020). Cardiovascular complications in COVID-19. Am J Emerg Med.

[16] Akintayo AA, Addo B, Soleye SO, Patel D, Ahmad A, Tongia S (2021). Diagnostic dilemma: COVID-19 related cardiomyopathy or peripartum cardiomyopathy?. J Cardiol Cases.

